# Fretting Fatigue Life Prediction of Dovetail Structure Based on Plastic Effect and Sensitivity Analysis of Influencing Factors

**DOI:** 10.3390/ma16093521

**Published:** 2023-05-04

**Authors:** Jianjun Zhou, Bowen Yang, Shuaiyuan Li, Junzhou Huo

**Affiliations:** 1 State Key Laboratory of Shield Machine and Boring Technology, Zhengzhou 450001, China; 2 School of Mechanical Engineering, Dalian University of Technology, Dalian 116024, China

**Keywords:** dovetail structure, fretting fatigue, life prediction, sensitivity factors

## Abstract

Micro relative sliding exists on the contact surface of the main primary equipment’s surface structures, resulting in serious fretting fatigue. The plastic effect causes serious fatigue to the structure under alternating loads. Existing fatigue life prediction models fail to fully consider the shortcomings of fretting and plastic effects, which causes the prediction results to be significantly different to real-lifeworld in engineering situations. Therefore, it is urgent to establish a fretting damage fatigue life prediction model of contact structures which considers plastic effects. In this study, a plastic fretting fatigue life prediction model was established according to the standard structural contact theory. The location of dangerous points was evaluated according to a finite element simulation. The cyclic load maximum stress value was compared with the fretting fatigue test data to confirm the error value, and the error between the proposed fretting fatigue life model and the test value was within 15%. Concurrently, we combined this with mass data analysis and research, as it is known that the contact zone parameters have an impact on fretting fatigue and affect the structural lifespan. With the help of ABAQUS, the fretting numerical calculation of the dovetail tenon model was carried out to analyze the sensitive factors affecting the fretting fatigue life of the dovetail tenon structure. By keeping the fretting load unchanged, the contact area parameters such as contact surface form, contact area width and friction coefficient were changed in order to calculate the fretting stress value, *σ_fretting_* and the dovetail structure was improved to extend its fretting fatigue life. Finally, it was concluded that fretting fatigue was most sensitive to the width and contact form of the contact area. In actual engineering design, multiple factors should be considered comprehensively to determine a more accurate and suitable width and form of the contact area. For the selection of friction coefficient, on the premise of saving costs and meeting the structural strength requirements, the friction coefficient should be as small as possible, and the problem can also be solved through lubrication during processing.

## 1. Introduction

**Figure 1:**
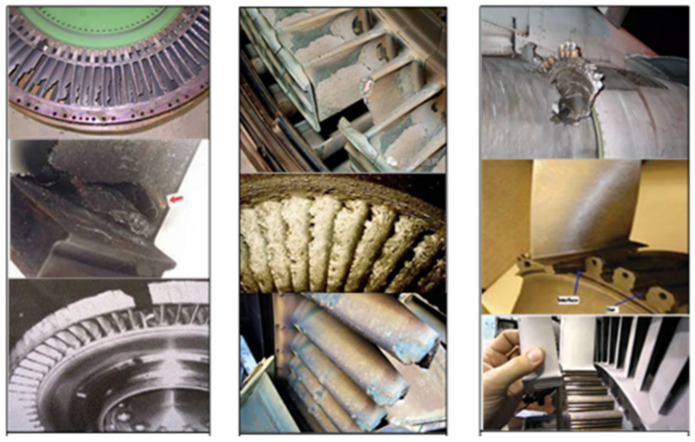
Fatigue damage diagram.

The extreme environmental complexity of major equipment requires the dynamic load of the main load-bearing structure to have strong mutation characteristics, which cause fatigue fretting wear. During actual service, serious damage and failure of key load-bearing components and other engineering problems occur, as shown in Figure 1. These dangerous parts can very easily produce micro-sliding, which leads to fretting wear, fretting fatigue, and other phenomena, resulting in a significant impact on fatigue life. Under the cyclic load, fretting fatigue refers to fretting wear, fretting fatigue, and other phenomena caused by the slight slip of structural members. Therefore, it is necessary to accurately predict the fretting fatigue life of structural members and understand the fretting damage mechanism on an in-depth level. The biggest difference between fretting fatigue and normal fatigue is the contact area of two components. Affected by the complex multiaxial load state in the contact area and the stress gradient at the edge of the contact area, the need to obtain accurate load values and the change trend of the contact area has become an important factor for scholars across the world when studying fretting fatigue. Studying fretting fatigue mainly involves the stress calculation under a multi-axial load to accurately predict the location of fretting crack initiation and fretting fatigue life under different external factors [1,2]. As shown in Figure 1, high and low circumferential cyclic fatigue due to stress concentration can be caused by the leaf root, leaf body, and other structural fatigue damage. The centrifugal tensile stress high-temperature pneumatic load causes a transient thermal strain long-time high temperature state, resulting in fatigue damage to the turbine curved blade body. Fretting fatigue causes fatigue damage at the drive shaft and turbine disk seat connection, the flange connection between the drive shaft and turbine disk or helical gear, and the dovetail-type connection of the blade wheel disk.

Fretting fatigue research covers the multi-axial load and severe stress distribution. Due to the limited harsh conditions of actual service conditions, it is difficult to predict the location of fretting crack initiation and fretting fatigue life. Therefore, many scholars have proposed multi-angle research methods and theories to solve the above problems, for example, based on empirical theory and experimental verification, the critical plane method [3,4], as well as the damage mechanics method [5,6], fretting specific parameter method [7], crack initiation and growth rate [8], etc. The critical plane method is the most commonly used stress–strain system with important damage parameters [9], followed by the KBM parameter method [10] and the FS parameter method [11]. The FP parameter method [12], SSR parameter method [13], and MSSR parameter method [14] are based on stress, while the L-parameter method is based on energy [15], as is the S-W-T parameter method [16]. The main classical continuous damage mechanical model is the Lemaitre damage model [17]. Zeng et al. [18] investigated the susceptibility of the steady-state properties of frictional interaction in preliminary conditions through digital analysis but without experimental verification. Mario et al. [19] proposed the computation of the maximum contact stress at the edge of the dovetail structure, based on the contact theory model. Lemoine et al. [20] analyzed the stress pattern in the contact zone of an aircraft blade under cyclic loading. The fatigue life theory based on stress–strain gradient cycles was established. Macdonald et al. [21] predicted the critical interface orientation and fretting fatigue life based on the stress–strain guidelines. However, normal stress and strain can only be used to predict primary life. The high stresses induced by fretting lead to the fatigue damage of the structure, which results in a plastic zone at the crack tip and rapid growth. Wahab et al. [22] predicted fatigue life by performing fretting fatigue tests. However, the model did not involve the effect of plasticity effects on fatigue. Han et al. [23] considered the effect of plasticity on estimating the free fatigue life, but only the onset of free fatigue was considered and the complete life expectation analysis, such as crack growth, was not investigated. Bhatti et al. [3] analyzed in depth the plastic effect of dovetails under frictional loading and the effects of different stress ratios but did not perform any experiments to verify the model accuracy. Pereira et al. [4] considered the combined influence of frictional and fatigue stresses on the structural parts but neglected to examine the effect of residual stress build-up on the lifespan of the structural parts. Sun et al. [24] investigated the effect of plastic transformation on the evolution of fretting fatigue damage, but the study did not consider the effects of plasticity on the lifespan of the material. Han et al. [25] assessed the effects of different conditions on crack clamping. Although crack tensioning could be analyzed, an in-depth study of fatigue life was lacking. Wu et al. [26] explored the plastic wear behavior of titanium alloys during friction and further analyzed the plasticity effect by monitoring the friction coefficient. Wang et al. [27] analyzed the effect of plastic deformation on the wear process using the FEM method, developed a computational model of cumulative plastic strain, and performed experimental validation. However, this model did not include the effects of plasticity on the fretting fatigue life. Therefore, it has a large margin of error.

Fretting fatigue depends on environmental factors. Relative humidity, corrosive media, vibration, contact pressure, friction, etc. may cause structural damage and cracking [28]. Sharma [29] measured the in-situ wear depth by micro-wear experiments under a normal load of 400 N. It was found that friction had a serious effect on the structure, with the least damage occurring when the friction coefficient was reduced by 50%. Sun [30] analyzed the effect of high temperature on the micro-action fatigue life of a dovetail structure, and established a micro-action fatigue life prediction model considering the criterion of micro-action fatigue crack sprouting failure based on circular plane contact theory. Liu [31] proposed an artificial neural network (HMNN) prediction method based on a fractal mechanism. The layers are proportional multi-axial fatigue, non-proportional multi-axial fatigue, notched fatigue and micro-motion fatigue. Based on the step-by-step construction of fatigue complexity, each layer can be used to evaluate the fatigue life of the previous layer. Mirsayar [32] investigated the effect of material anisotropy on the fatigue crack propagation (FCG) behavior of additively manufactured materials by establishing an energy-based criterion. It was found that material anisotropy, caused by both tectonic orientation and T-shaped stresses, plays a significant role in the FCG behavior of 3D printed components.

In summary, because fretting wear is not easy to observe during crack initiation and fretting damage generally occurs in the contact zone or the edge of the contact zone, which is also difficult to observe, many fatigue damage studies focus on the fracture mechanics method of predicting crack growth life, which is not applicable to predicting fretting fatigue lifespan. The prediction of free fatigue life using the continuous cumulative damage theory needs to consider many factors. Due to the complexity of the structure, the prediction of fretting fatigue initiation and propagation still lacks in-depth theoretical research. Therefore, in order to accurately control the service life impact of fretting fatigue, it is a key research necessity to establish a fretting fatigue life prediction model according to theoretical analysis and the fretting fatigue test.

## 2. Materials and Methods

Due to the special characteristics of the aero-engine dovetail structure and the complexity of multi-axial loading, there is no valid theoretical model to estimate micro-motion fatigue life (initial and total life). Based on the fretting fatigue contact theory and the progressive damage model, the effect of forming effects on the dovetail structure is considered. In order to provide accurate estimates, the research objectives of this paper are: (i) to develop a face contact fretting fatigue life estimation model using the aero-engine dovetail structure as the research object; (ii) to evaluate the validity and applicability of the estimated model by combining theoretical analysis, finite element simulation, and fretting fatigue experiments; and (iii) to analyze the sensitivity of dovetail structure parameters based on the micro-action fatigue mechanism with reference to the stress distribution law and the objective of minimizing micro-action stress.

### 2.1. Establishment of the Fretting Fatigue Life Prediction Model

Fatigue damage is caused by the severe expansion of tiny fine lines and initial defects. The internal damage variables are introduced by considering the damage variables as internal. Additionally, in order to target different material structural damage, the geometry state variable *D* is introduced [33].

The damage variable *D*, represented as the presence of tiny defects in the unit volume of the material, is shown in Equation (1).

(1) $$D=\frac{A-\tilde{A}}{A}$$

The accumulated damage *D* is not directly accessible by existing means, so it needs to be calculated using the measured effective area *A*. Therefore, the link between the elastic modulus *E* and the damage *D* is established by transformation, and finally, the elastic modulus variation law is obtained experimentally.

Using Hooke’s law (*σ* = *F*/*A*) and cumulative damage theory, the equation is established using the indirect variable of area *A*. The effective stress $\tilde{\sigma }$ is:

(2) $$\tilde{\sigma }=\frac{F}{\tilde{A}}=\frac{\sigma }{1-D}$$

We proposed the concept of fretting stress *σ_fretting_* based on the structure:

(3) $$\sigma_{fretting}=\sigma_{0}+2p_{h}\sqrt{\frac{\mu F_{T}}{F_{N}}}$$

*p_h_* is the maximum load in the contact zone, *μ* is the fretting coefficient of the contact surface, *F_T_* is the tangential load amplitude of standard sample and contact pad, *F_N_* is the normal load, and *σ*_0_ is the peak value of axial stress.

In 2014, Aditya A. Walvekar of Purdue University introduced fretting stress based on theoretical research and experimental verification, *σ_fretting_*, and proposed a damage evolutive formula of fretting fatigue, in which the damage parameter *σ_R_* is a material parameter, which is the fatigue damage parameter of materials under cyclic loading. *σ_R_* is the material under fully reversed loading of the resistive stress, with a stress ratio of −1, and is a function of the average stress. In the non-damaged state, *D* = 0 and *N* = 0, and under the completely damaged state, *N* = *N_f_* and *D* = *D_crit_*; the derivation is as follows.

(4) $$\frac{dD}{dN}={\left[\frac{\sigma_{fretting}}{\sigma_{R}(1-D)}\right]}^{m}{\left(\Delta \varepsilon \right)}_{p}^{w}$$

In order to obtain a more accurate fretting fatigue life model of dovetail joints, it is necessary to consider the significant plastic strain deformation in the contact zone caused by the combined action of fretting and fatigue stress. The plastic effect not only affects crack generation and expansion but also leads to contact area expansion for fretting fatigue. However, the basic contact zone expansion is mainly attributed to increasing frictional work generated by the relative sliding of the contact surfaces over time, due to the constant action of the alternating load, the plastic deformation of the contact surface, and the deep penetration of the active surface into the driven surface. Thus, in this paper, the influence of the plasticity effect on fretting fatigue is characterized by the maximum plastic strain at the edge of the contact zone during the process, from the undamaged state to the fully damaged state of the specimen. This assumes that the cumulative prediction model of fretting fatigue damage considers plasticity, as shown in Equation (5):

(5) $$N_{f}={\left(\frac{\sigma_{R}}{\sigma_{fretting}}\right)}^{m}\left[\frac{1}{m+1}-\frac{{\left(1-D_{crit}\right)}^{m+1}}{m+1}\right]{\left(\Delta \varepsilon \right)}_{p}{}^{w}$$

where *D_crit_* is the critical damage parameter. (Δ*ε*)_*p*_ is obtained from experimental measurements and is expressed as the maximum plastic strain value that reaches the critical damage in the test. Based on the above formula derivation, the fretting fatigue damage accumulation model considering plastic effect is finally obtained by combining the contact area load solution, fretting fatigue test data, fitting parameters, etc. The biggest advantage of this model is that the influence of fretting stress, fatigue stress and plastic effect is comprehensively considered, so that the prediction results are more accurate.

We improve the progressive damage method in order to solve the problem of incomplete contact. A generalized stress intensity factor was introduced to specifically analyze the stress state at the edges of the asymptotic contact, and the asymptotic technique was successfully applied to predict fatigue life. Thus, the contact theory can determine the maximum contact stress *P_h_* of the structure, and the remaining unknown parameters need to be calculated using experimental data.

The contact load *P*(*t*) is formulated:

(6) $$p\left(t\right)=\{\begin{array}{l} \begin{array}{cc} \;\;\;\;\;\frac{K_{N}}{\sqrt{t}} & \begin{array}{cc} \mathrm{if} & t/a_{round}\gg 1 \end{array} \end{array}\\ \begin{array}{ccc} \frac{3K_{N}\sqrt{t}}{a_{round}} & \;\mathrm{if} & 0<t/a_{round}\ll 1 \end{array} \end{array}$$

(7) $$K_{N}=\frac{-2E*\sqrt{a_{round}{}^{3}}}{3\pi R}.$$

(8) $$p\left(x\right)=\frac{3K_{N}}{4\sqrt{a_{round}^{3}}}\left[2\sqrt{xa_{round}}+\left(x-a_{round}\right)\ln\left|\frac{\sqrt{a_{round}}-\sqrt{x}}{\sqrt{a_{round}}+\sqrt{x}}\right|\right]$$

where *a_around_* is the radius of the load contact zone, *t* is the contact edge, *R* is the edge radius, and E* is the modulus of elasticity.

### 2.2. Fretting Fatigue Simulation Analysis and Dangerous Point Evaluation

The ABAQUS is used to simulate and analyze the standard specimen. The analysis was performed in ABAQUS Explicit. The plastic model was used for finite element analysis. The Johnson–Cook model was used for damage. The Johnson–Cook model is shown in Equation (1), and the specific parameters are shown in Table 1.

(9) $$\sigma =\left[A+B{\left(\varepsilon_{p}\right)}^{n}\right]\left[1+C\ln\left(\frac{{\dot{\varepsilon }}_{p}}{{\dot{\varepsilon }}_{0}}\right)\right]\left[1-{\left(\frac{\theta -\theta_{room}}{\theta_{melt}-\theta_{room}}\right)}^{m}\right]$$

In the equation, *A* is the yield strength under quasi-static conditions. *B* is the strain hardening parameter. *C* is the strain rate hardening parameter. *m* is the thermal softening parameter. *n* is the is the process hardening parameter. *ε*_0_ is the quasi-static strain rate. *θ_melt_* is the material melting point. *θ_room_* is the room temperature.

**Figure 2:**
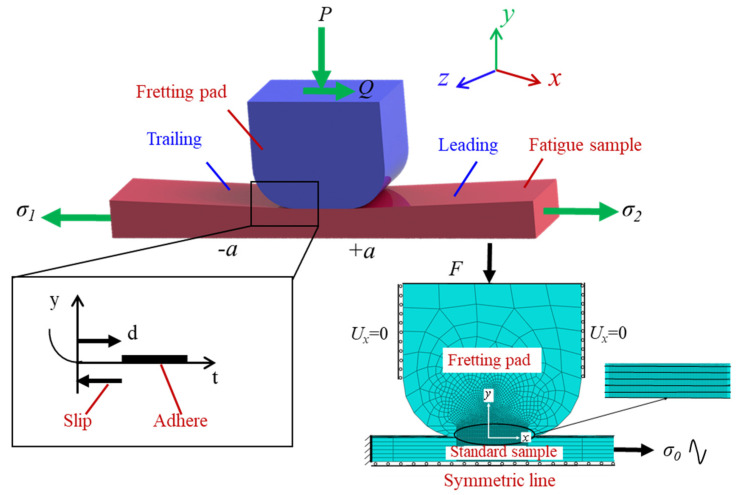
The finite element model of fretting.

Considering the symmetry of the model, take one half of the model and apply the load under the following conditions: the left end of the standard specimen is subject to a fixed constraint and the lower end is subject to a symmetrical constraint. The left and right ends of the inching pad limit displacement in the x direction. Apply the sinusoidal cyclic axial load to the right end of the standard test piece, and apply the normal load to the upper end of the fretting pad. The contact area finite element meshing element is 100. The augmented Lagrange method was used for the finite element analysis of the contact. The finite element model is shown in Figure 2.

**Figure 3:**
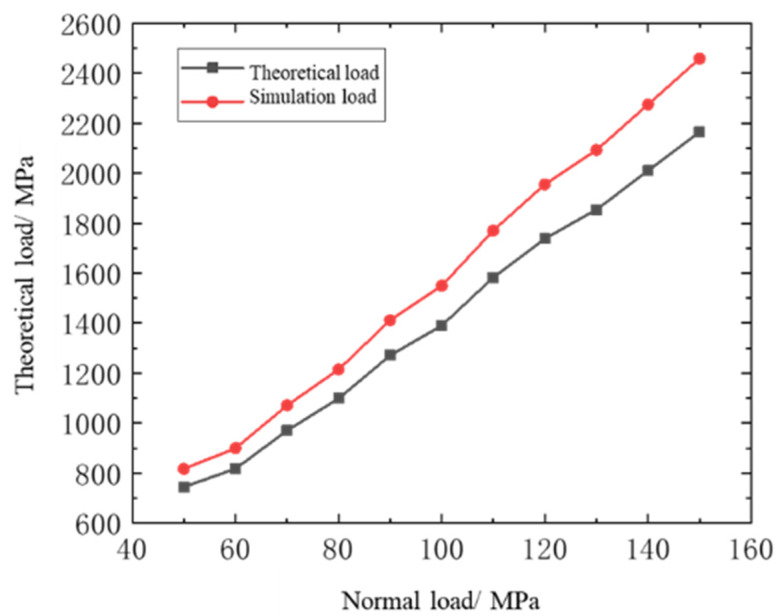
Comparison of simulation and theoretical results of contact stress.

Keeping the cyclic axial load at 300 MPa constant and taking the normal load as 45–150 MPa, the numerical analysis of fretting was performed on the standard specimen and the fretting pad to obtain the contact stress *P*_0_ corresponding to different normal loads. The calculated theoretical result, *P*_1_, was compared to the finite element simulation and the theoretical calculated analysis; the error was within 13%, as shown in Table 2, and the fitted curve is shown in Figure 3. The accuracy of the numerical analysis of the fretting force of the standard specimen with ABAQUS is proven.

The deviation between the theoretical and simulated loads may be mainly due to the friction of the contact area, the deviation of the actual size modeling, the mesh division as required in the simulation, the elastic–plastic damage theory, the force area of the contact area for different load forces, and the rounding in the calculation. We made an accurate comparison to ensure the accuracy of the simulation and that the error is within the acceptable range.

**Figure 4:**
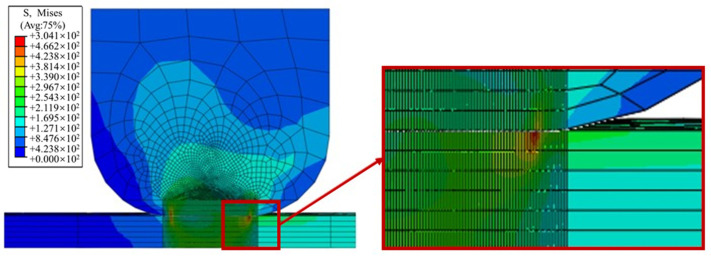
Stress nephogram (normal load is 45 MPa) and partial enlarged drawing of the trailing edge of the contact area.

In Figure 4, the stress cloud diagram corresponding to the normal load of 45 MPa is shown. It can be observed that different normal loads will not change the weak position of the structure. Therefore, it is possible to determine that the danger point for fretting fatigue occurs at the resulting joint.

## 3. Results

### 3.1. Fretting Fatigue Test and Analysis

In the fretting fatigue damage accumulation model considering the plastic effect and the contact area load solution, the unknown parameters in the assumed model are obtained from the fretting fatigue life data under different loads. Therefore, the fretting fatigue test is performed on the standard sample.

#### 3.1.1. Test Materials and Samples

**Figure 5:**
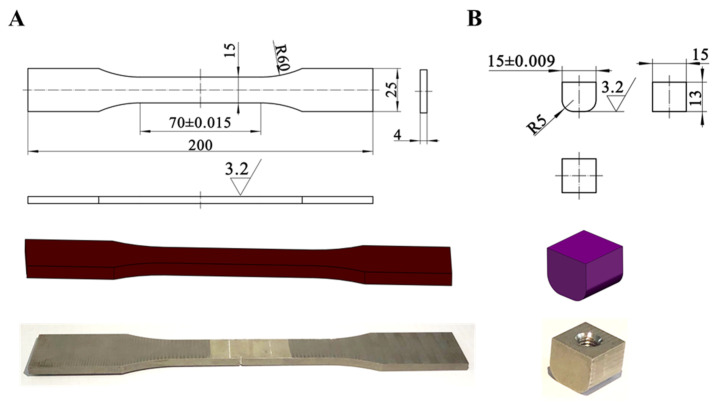
Schematic diagram of fretting test sample (**A**) standard sample; (**B**) fretting pad.

The test material is a commonly used nickel base alloy, DZ125. DZ125 is a nickel base precipitation-hardening and directionally solidified columnar alloy that contains elements. It has good overall performance and outstanding fatigue properties. Using the data findings from uniaxial tensile tests, it was calibrated according to the mechanical principles. The property test is shown in Table 3, and the standard samples and fretting pads of the fretting tests are shown in Figure 5.

#### 3.1.2. Test Equipment

**Figure 6:**
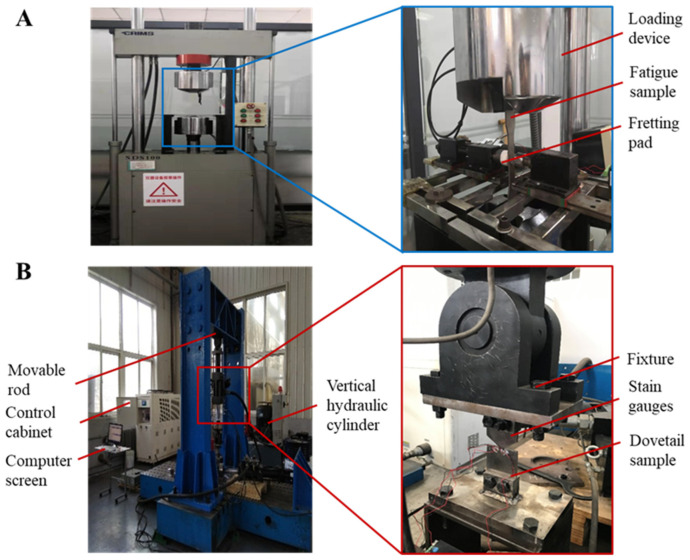
Fretting fatigue test system. (**A**) Standard sample test system and (**B**) Dovetail fatigue test system.

According to the test scheme and requirements, the appropriate test equipment was selected. The fretting fatigue test system independently designed by the team of the Dalian University of Technology is used in this test. The equipment and instruments required for the fretting fatigue test include: one SDS100 electro-hydraulic servo fatigue test machine; one control system; one computer; one gateway node; and one normal loading device (consisting of four parts: platform, guide rail, sliding module, and tension sensor). As shown in Figure 6, the fretting fatigue test system of a standard specimen is adopted.

#### 3.1.3. Test Plan

According to the overall technical scheme, the fretting fatigue test of the standard sample is divided into two parts. The test selects six standard samples with different load points, and each load point is subject to two groups of tests, which are divided into displacement control conditions and load control conditions, according to the load. The first part requires the measurement of the critical damage parameter *D_crit_* of the sample by changing the elastic modulus and obtaining the maximum plastic strain (Δ*ε*)_*p*_. Displacement control is adopted for test loading control. In the second part, fretting fatigue tests under different cyclic loads are carried out and their fatigue lives are recorded. Combined with the theoretical analysis results, a fretting fatigue life prediction model is established. Load control is adopted for testing loading control.

The fatigue life under different loading conditions was obtained, and a fatigue life model was established through analytical data. Strain plates were pasted on one side of the contact area between the standard samples and the micro pad, and the strain at the edge of the contact area was monitored in real-time. A constant normal load of 100 N was applied with a stress ratio of 0.1 and peak levels of 1.2 × 10^4^ N, 1.5 × 10^4^ N, 1.8 × 10^4^ N, 2.1 × 10^4^ N, 2.4 × 10^4^ N, and 2.7 × 10^4^ N cyclic sinusoidal axial loads. The load parameters are shown in Table 3. The lives of the standard specimens under different loads were monitored. The test was terminated when a strain mutation occurred at the contact area of the standard specimen and the fretting pad under the fretting load. The fretting fatigue test resulted from the standard specimen.

#### 3.1.4. Fretting Test Results and Analysis

**Figure 7:**
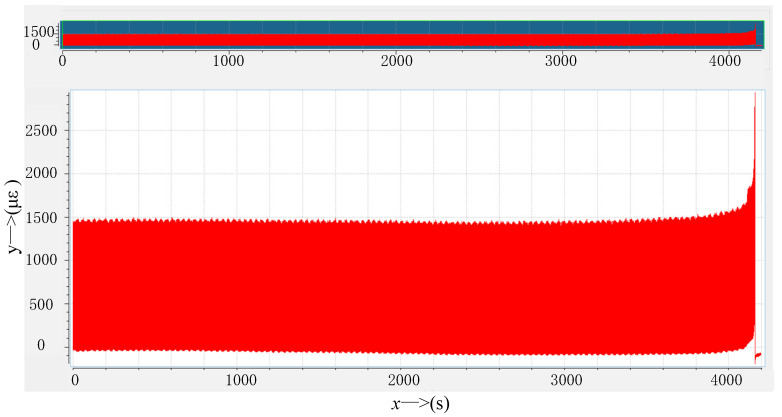
Diagram of monitoring strain.

In this paper, a method of monitoring the strain at the lower edge of the contact zone in real time was used to determine the generation of cracks. This was achieved by attaching strain gauges to the side of the lower edge of the contact zone where the standard sample was in contact with the fretting plate and connecting strain nodes to monitor the strain changes at the lower edges of the contact zone during the fretting fatigue test. As the test time increases, its strain slowly increased until the strain grid was sheared at a certain point and the strain suddenly increased to infinity. At this point, cracks appeared on the sample and fractured rapidly. This moment is considered to be the fatigue life of the sample, as shown in Figure 7.

**Figure 8:**
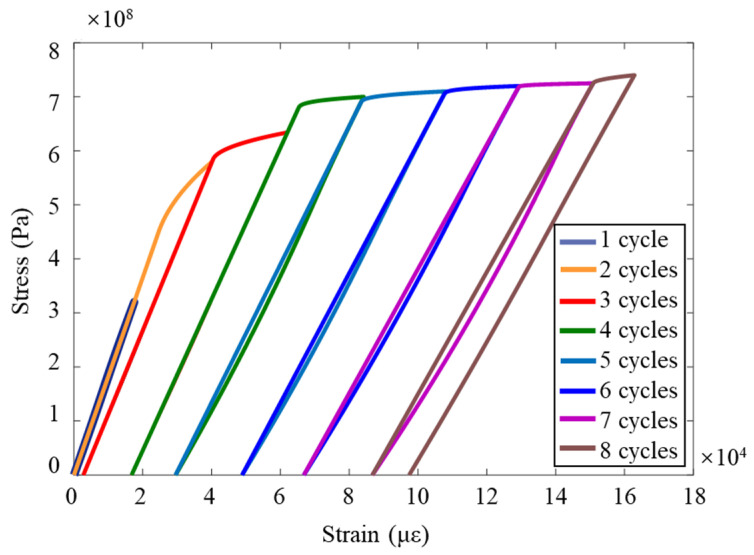
Stress–strain curve of elastic modulus change test.

Although many researchers use the mechanics of damage predict lifespan, they usually assume that in the preliminary state, the material has no deficiency, and so *N* = 0 and *D* = 0. In the final state, the crack occupies the entire surface of the representative volume element at the time of fracture *N* = *N_f_* and *D* = 1. However, according to the long-term fretting test and theoretical experience, before the fracture of the sample, due to the atomic debonding in the residual resistance area, the crack will occupy a certain area of the representative volume unit and suddenly break. Therefore, in the end state, *D* is defined as *D_crit_* instead of 1. The critical damage *D_crit_* at the time of fracture can be calculated by measuring the change of the elastic modulus of the material during the test. This method is the elastic modulus change method proposed by Lemaitre. Accurate strain measurement is very important for obtaining *D_crit_*, so strain gauges are installed in the constant cross-sectional area of the specimen. The test is carried out under the conditions of displacement control. In each cycle, the displacement gradually increases until plastic deformation occurs. The displacement is then reduced until the force (the stress within the specimen) returns to zero. In the next cycle, the displacement further increases, which gradually produces more plastic deformation, and then returns to the state where the force is zero. This process is repeated until the specimen breaks. Figure 8 depicts the stress–strain diagram of each cycle of the test and calculates the elastic modulus according to the slope of the unloading curve. In the expected case, the elastic modulus decreases with the increase in plastic deformation (that is, the damage to the specimen is increasing). Table 4 shows the decrease in the elastic modulus with the increase in damage. The critical damage value *D_crit_* at fracture is 0.382.

As shown in Figure 8, it can be seen that the number of cycles increases and the strain value increases slowly, indicating the accumulation of plastic strain. Assuming that the plastic strain of the same material before fracture is basically constant, the maximum plastic strain during cyclic dislocation loading can be utilized to obtain the maximum plastic strain generated during the whole process from the undamaged state to the fracture of the test sample, where (Δ*ε*)_*p*_ is 9.73 × 10^−2^. The fretting fatigue test of the standard sample was carried out through the test system. The crack fracture diagram of the sample after the test obtained the sample life and fretting stress under different axial loads; the σ fretting to obtain the fretting stress *σ_fretting_* relationship between fretting and fatigue life, *N*, is shown in Table 5.

**Figure 9:**
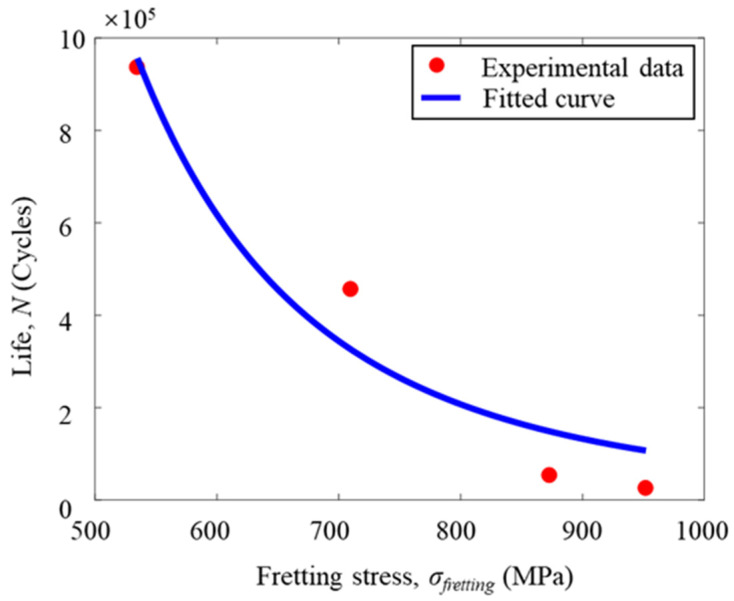
Relationship between fretting stress to life.

**Figure 10:**
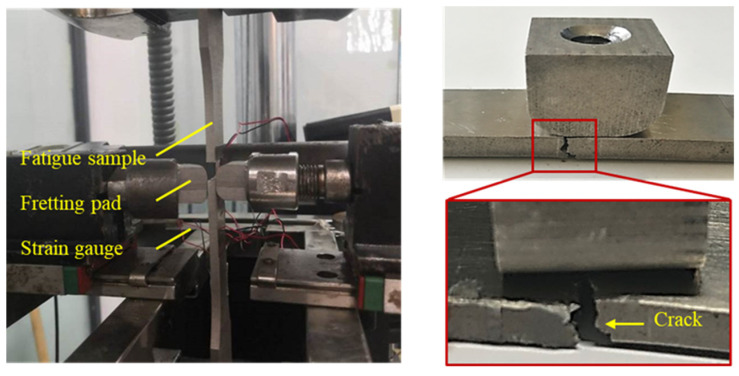
Fracture diagram of standard sample.

Synthesizing all the data and fitting according to the hypothetical theoretical model to determine the value of the unknown parameter, the final result is *m* = 3.8, *σ_R_* = 6774.58, *w* = −2.49. The fitting curve of test results is shown in Figure 9 below:

The schematic diagram of the fractured sample is shown in Figure 10. It can be seen that the strain value at the trailing edge of the contact area slowly increases with time as the number of fretting load cycles grow, signifying the accumulation of plastic strain. At a certain point, the strain suddenly changed in value and the sample fractured rapidly. It is obvious that the fatigue occurs near the contact zone and not at the change in the size of the standard sample, indicating that the fatigue is caused by the high stress gradient in the contact zone, which is consistent with the basic characteristics of a flying edge and proves that the fatigue is due to the flying edge and not otherwise. This proves the accuracy of the fretting fatigue test and ensures the validity of the test data, as well as the validity of the cumulative fretting fatigue life prediction model of successive damages considering the plastic effect established from the test data.

To verify the accuracy of the model based on the data of Test 2 and Test 5, as shown in Table 6, the theoretical prediction value of fretting fatigue life was compared with the test value, and the error was within 15% of the reasonable error. Therefore, the accuracy of the surface contact fatigue life prediction model based on the plastic effect is proven.

### 3.2. Fatigue Test and Analysis of Dovetail Structure

The fretting fatigue life prediction model considering the plastic effect is also suitable for dovetail structures.

The dovetail structure test sample is designed and the fretting fatigue test is conducted according to the standard sample materials and test equipment.

#### 3.2.1. Determining the Dangerous Location of a Dovetail Structure

Peak values are applied to the dovetail model tenon 8.58 × 10^3^ N, 1.18 × 10^4^ N, and 1.35 × 10^4^ N cyclic axial load, and the mean contact stress CPRESS and the Mises equivalent stress S in the contact zone of a dovetail structure are analyzed in order to identify the structural danger points.

**Figure 11:**
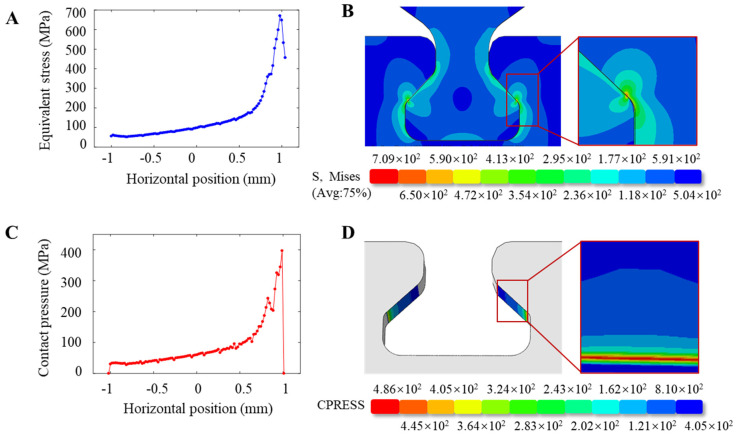
Stress nephogram distribution of dovetail model contact zone; (**A**) Mises equivalent stress force analysis of the dovetail model contact zone; (**B**) the amplification of Mises equivalent stress S nephogram and the contact zone; (**C**) the force analysis of contact stress in the contact area of dovetail; (**D**) the contact stress nephogram and contact area enlargement.

As shown in Figure 11, the contact stress CPRESS and the equivalent Sand stress distribution clouds are present in the contact zone at the 50th cycle. The red part is the highest concentration point in the tenon–tenon contact zone, and the maximum value of Mises equivalent stress S is 709.2 MPa. The maximum value of the contact stress CPRESS reached 486 MPa and generally showed a monotonic increasing trend from the front edges of the contact area to the rear edge of the contact zone. Under cyclic axial load, the amplitude of relative displacement increases near the contact zone rear edge. The work generated by the fretting between the mortise and tenon contact area increases, causing an extension of the stress gradient at the trailing edge of the contact area (Horizontal position → 1).

#### 3.2.2. Fretting Fatigue Test of Dovetail Specimen

**Figure 12:**
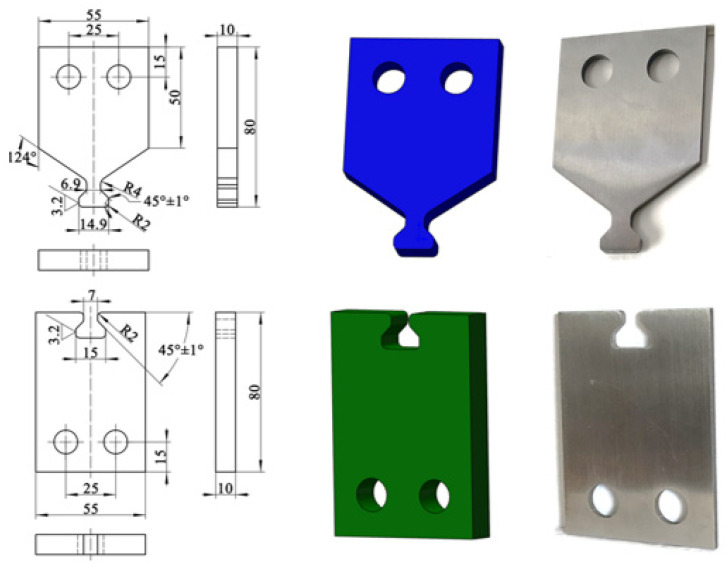
Model dimensions and physical drawing of the dovetail.

T The fretting fatigue life of dovetail test with various loading methods was found. The model based on the standard specimen was analyzed and verified to be applicable to the dovetail specimen. The dovetail samples were combined, and strain gauges were attached around the bottom edge of the dovetail contact zone of the dovetail samples. The strain change trend in the dovetail contact area was monitored in real-time in the experiment. The model dimensions and the physical drawing of the dovetail are shown in Figure 12. The sinusoidal cyclic axial load is applied with a stress ratio of 0.1 and a frequency of 10 Hz to the dovetail, and the peak values are 8.58 × 10^3^ N and 1.18 × 10^4^ N. We then undertook the real time monitoring and recording of the strain change trend, actuator displacement, and dovetail sample life. The experiment ended when the strain in the touch zone of the dovetail specimen changed abruptly under the cyclic load. Finally, the fretting fatigue test results of the dovetail specimens were obtained.

Under working conditions, there are mainly two kinds of cyclic loads borne by aeroengine turbine blades and discs: one is a centrifugal load of blades, the other is a vibration load of blades. The tenon slides up and down in the mortise under the action of centrifugal force. Under the vibration load action, the tenon swings left and right in the mortise. However, the largest swing amplitude caused by this vibration load is at the upper end of the blade, and the mortise and tenon fit. If the vibration load increases to the moving tenon, the blade will break due to ordinary fatigue, and fretting fatigue is not the main problem. Therefore, only the fretting fatigue between the dovetail and the mortise under centrifugal load is considered here.

#### 3.2.3. Fretting Fatigue Test Results of Dovetail Specimens

**Figure 13:**
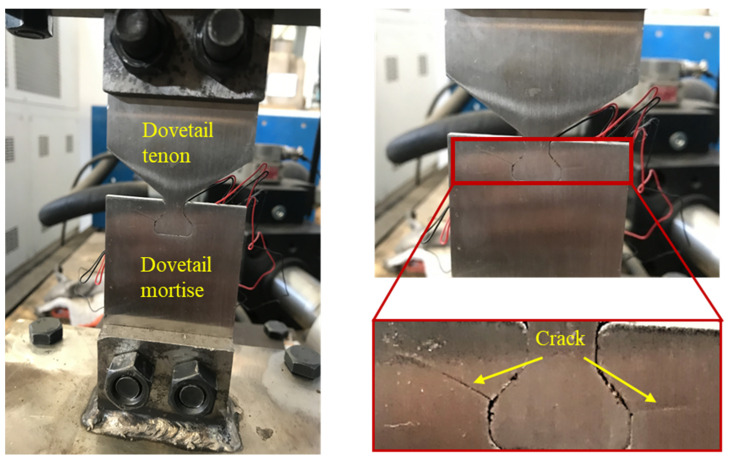
Dovetail sample crack (the peak value of cyclic axial load is 1.18 × 10^4^ N).

Table 6 shows the fretting fatigue test results of dovetail with different load conditions, from which the life of the specimens at axial load is calculated. The peak axial load of the real object after the fracture of the sample (Figure 13) is 1.18 × 10^4^ N. It can be seen that fatigue failure occurs around the contact zone, suggesting that fatigue is caused by the high-stress gradients in the contact zone. The fatigue of the dovetail structure is caused by fretting fatigue due to the relative sliding between dovetail tenon and mortise. Therefore, the test results can be used to confirm the suitability of the model.

The damage cracks produced by the dovetail specimens are analyzed in Figure 13. According to Equation (3), the micro-motion stress in the micro-motion test of the dovetail specimen was calculated, and then the micro-motion fatigue life prediction model of Equation (5) was used to predict the life and the results were compared with the test life, as shown in Table 7. It can be concluded that the theoretical calculation structure is relatively conservative, and the theoretical lives are all smaller than the experimental lives, but the errors are within the range of 12%. Therefore, the life prediction model is based on the fatigue life prediction of the dovetail structure specimens.

The contact area of the trailing edge of the contact area, the amount of wear of the tiny slip, the error of the equipment in the actual test process, etc., will cause uncertainty in the load loading, and the tiny error of the micro-motion stress will have some influence on the fatigue life prediction process. In addition, our actual engineering applications tend to use more conservative design ideas in order to ensure the safe and reliable performance of the structure.

## 4. Sensitivity Analysis of Structural Parameters

### 4.1. Sensitivity Analysis of Factors Affecting Fretting Fatigue Life of the Dovetail Structure

Dovetail structure connection and assembly conditions are complex. Contact geometry, loading conditions, surface treatment, etc., will affect the failure mode and damage levels of the blade disk interface. By selecting three main characteristic parameters as optimization variables, taking the stress distribution law as a reference, and taking the fretting stress, *σ_fretting_*, the goal of minimizing fretting is to improve the dovetail mortise structure by using the finite element analysis software ABAQUS (CAE2018, DASSAULT SIMULIA, Provision, RI, USA).

**Figure 14:**
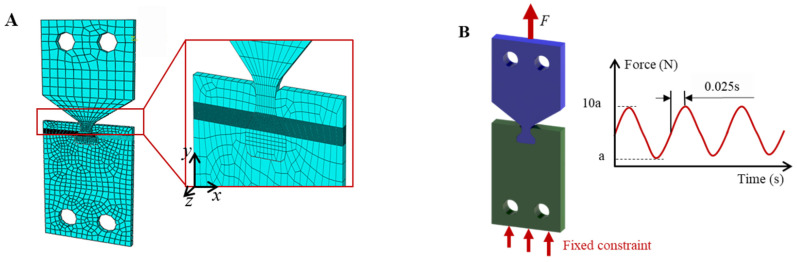
Dovetail structure finite element model (**A**) Dovetail meshing; (**B**) Boundary conditions and loading.

Due to the specificity of the contact area load calculation for the dovetail structure, it is important to obtain the exact contact area load through the direct theoretical model. Thus, the finite-element simulation tool ABAQUS (CAE2018, DASSAULT SIMULIA, Provision, RI, USA) was used to solve the contact area load calculation. Due to the specificity of the contact area load calculation for the dovetail structure, it is important to obtain the exact contact area load through the direct theoretical model. Thus, the finite-element simulation tool ABAQUS (CAE2018, DASSAULT SIMULIA, Provision, RI, USA) was used to solve the contact area load. The eight-node linear element C3D8R was used to conduct numerical analysis in combination with the “master–slave” interface algorithm of the ABAQUS finite element program. The dovetail structure finite element model was established according to the processing sample, as shown in Figure 14A. The lower end was fixed, and the maximum amplitude of upper end loading was a 1.35 × 10^4^ N, 1.18 × 10^4^ N, and 8.6 × 10^3^ N cyclic axial load. The dovetail structure was divided into meshes, with the non-significant regions of the model roughly divided and the contact regions were subdivided. During the mesh aggregation and analysis, the number of grids in the contact area were 50, 60, 70, 80, 90, 100, 110, 120, 130, 140, and 150, and the contact stress CPRESS and Mises equivalent stress S were obtained through numerical analysis. The contact stress CPRESS and the equivalent stress S of Mises under different grid sizes can be used for comparative analysis. The contact area element meshing element is 100. The analysis was performed in the ABAQUS Explicit. The contact surface simulation sets the friction force to 0.8. The finite element simulation is loaded with a sinusoidal fatigue cyclic load. The finite element model of the dovetail structure is shown in Figure 14.

### 4.2. Form of Contact Surface

**Figure 15:**
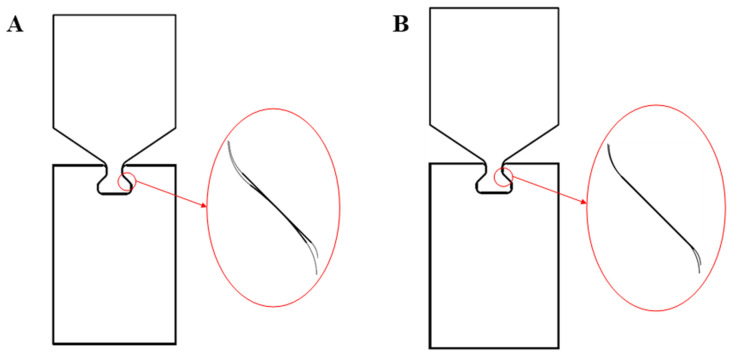
Dovetail models with two different contact forms: (**A**) arc dovetail profile (line to surface contact); (**B**) plane dovetail profile (surface to surface contact).

For the study of contact surface forms, the main purpose is to compare the contact forms of a circular tenon surface and a flat tenon surface with the tenon. The initial contact form between the circular tenon surface and the mortise plane is a line surface contact, while the initial contact form between the planar tenon surface and the mortise plane is surface contact. The size of other parts should be kept unchanged, and only the form of the tenon surface should be changed, as shown in Figure 15, which is a comparison of two different contact forms.

The different contact forms will lead to different states of the contact zone subjected to fretting load and affect the distribution of stress–strain. By means of ABAQUS, parameters are set, applying the same constraint and loading to the two models with different contact forms; the same contact area parameters are set, numerical analysis is conducted, and the Mises equivalent stress S is analyzed, along with contact stress and fretting stress *σ_fretting_* contact surface forms of fretting fatigue between dovetail models with different contact surface forms.

Holding the cyclic axial load at a constant peak value of 1.13 × 10^4^ N, a comparison of the Mises equivalent stress S and contact stress distribution diagrams for the dovetail contact areas of the two different contact forms subjected to the tearing load is made, and thus the tearing stress *σ_fretting_*, was carried out.

**Figure 16:**
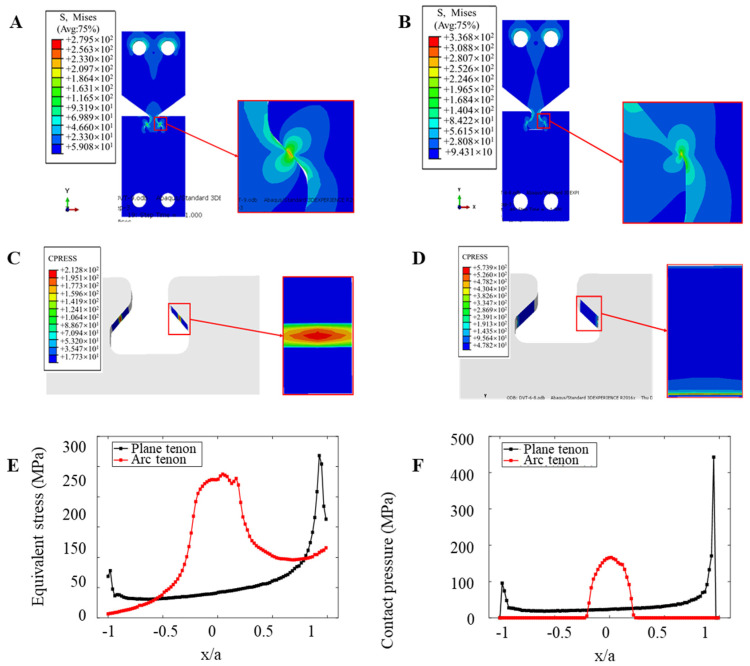
Comparison diagram of equivalent stress S and contact stress of two different forms of tenon profiles: (**A**) Mises equivalent stress S distribution of arc tenon profile; (**B**) Mises equivalent stress S distribution of plane tenon profile; (**C**) contact stress distribution of arc tenon profile; (**D**) contact stress distribution of plane tenon profile (**E**) Mises equivalent stress value of two different forms of tenon profiles; (**F**) contact stress value of two different forms of tenon profile.

As shown in Figure 16, qualitatively, although the contact forms are different, under the action of fretting load, the maximum values of the equivalent stress S and the contact stress of the mortise Mises appear at the edge of the contact area. The comparison diagram of the Mises equivalent stress S and contact stress distribution of two different types of tenon profile is shown in Figure 16 (the size of the contact area between the planar tenon profile and the mortise is taken as the abscissa in the figure). From a quantitative perspective, under the same fretting load, the maximum Mises equivalent stress generated by the contact between the arc tenon profile and the mortise is 57.3 MPa lower than the maximum Mises equivalent stress generated by the contact between the plane tenon profile and the mortise. The maximum contact stress was 361.1 MPa lower than the maximum contact stress generated by the contact between the flat tenon face and the mortise, and the final fretting stress value obtained, *σ_fretting_*, was 493.563 MPa lower than the fretting stress value, *σ_fretting_*, generated by the contact between the flat tenon face and the mortise.

It can be concluded that the variation of the contact zone form of the dovetail model does not affect the stress distribution and the location of the danger points of the structure under the fretting load. However, the fretting stress value of the linear surface contact form produced by the circular tenon to tenon contact is smaller than that of the surface contact form produced by the planar tenon to tenon contact, and the fretting fatigue life of the model is longer. Therefore, under the premise of satisfying the assembly requirements and structural strength requirements, the two matched structural members should adopt the form of line-to-surface contact as much as possible. Since only the axial fretting fatigue behavior of a pair of reduced diameter dovetail models is considered here, and no other factors are taken into account, a more suitable contact surface form should be selected according to various conditions in engineering practice.

### 4.3. Width of Contact Area

**Figure 17:**
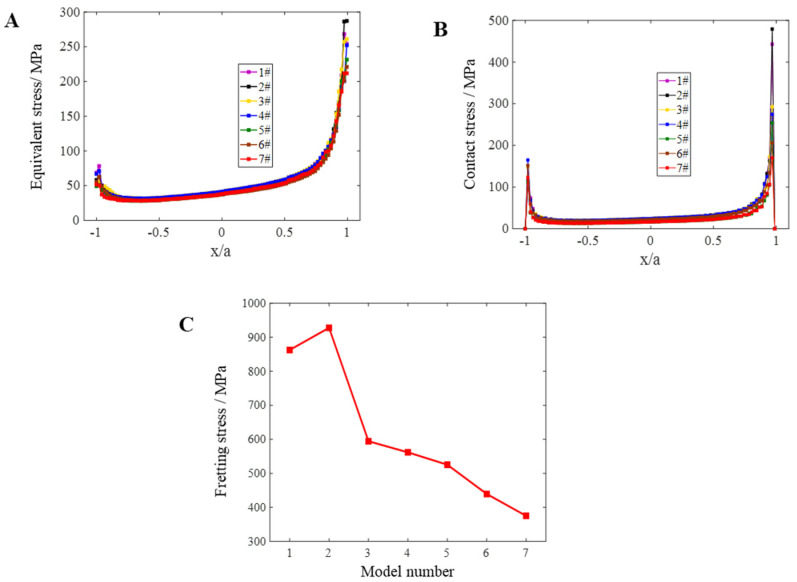
Fretting stress of models 1–7 under the same load *σ_fretting_* comparison: (**A**) Mises equivalent stress S value of models 1–7; (**B**) contact stress value of models 1–7; (**C**) fretting stress comparison under the same load conditions *σ_fretting_*.

In addition to the contact form, the width of the contact area also affects the distribution and gradient of the load in the contact area. During the research on the influence of the width of the contact surface on fretting fatigue, the dovetail model will keep the size of other parts unchanged and only change the size of the fillets near the contact area and the width of the contact area. As shown in Figure 17, the model diagram is shown. Table 8 details the width dimensions of the corrected fillet and contact zone for each model.

The different contact zone widths lead to different states of the contact zone subject to frictional loads, which in turn affect the distribution of stresses and strains. By setting parameters with the help of numerical analysis software ABAQUS, parameters are set, the same constraints and loads are applied to each model with different contact area widths, the same contact area parameters are set, numerical analysis is conducted, and the contact stress is analyzed, followed by the Mises effect force S and fretting stress between the dovetail models under different contact area widths and the *σ_fretting_* influence of the contact zone width on fretting fatigue.

Holding the peak cyclic axial load of a 1.13 × 10^4^ N constant, the Mises effect stress S, the comparison of contact stress profiles, and the comparison of free stress *σ_fretting_* in the contact zone of the dovetail groove under different contact zone widths were carried out.

Figure 17 only lists the Mises equivalent of the stress S nephogram and contact stress nephogram of models 1 and 2, but not all models. This is because qualitatively, as long as the contact is plane contact, there is not much difference in its stress distribution under the action of fretting load. The maximum values of the equivalent force S and contact stress S in the tenon occur at the edges of the contact zone and do not vary. Therefore, only the stress nephograms 1 and 2 are shown here as representatives. The quantitative analysis shows that when the width of the contact zone decreases from 4.343 mm to 4.302 mm, the maximum Mises equivalent stress S and the maximum contact stress and fretting stress *σ_frettin_*_g_ values increase slightly, but on the whole, with the decrease in the contact zone width, the maximum Mises equivalent stress S, the maximum contact stress and the fretting stress *σ_fretting_* values tend to decrease. In general, keeping other parameters unchanged, the width of contact zone is reduced from 4.343 mm to 4.095 mm, and the reduction amplitude is only 248 μm. In the case of fretting load, the maximum Mises equivalent stress S decreases by 75.286 MPa, the maximum contact stress decreases by 308.994 MPa, and the *σ_fretting_* value decreases by 552.745 MPa; moreover, the fretting stress directly affects the fretting fatigue life, so it can be seen that fretting fatigue is very sensitive to the change of contact zone width. Separately, as the width of contact zone gradually decreases, the maximum Mises equivalent stress S, maximum contact stress, and fretting stress *σ_frettin_*_g_ is shown in Table 9. It can be seen that when the width of contact zone decreases from 4.261 mm to 4.095 mm from models 3 to 7, and the corresponding maximum contact stress and fretting stress *σ_fretting_* reduction in fretting value has little difference. In models 2 and 3, when the width of contact area is reduced from 4.302 mm to 4.261 mm, the corresponding maximum contact stress and fretting stress *σ_fretting_* value decreased abruptly. In conclusion, when the minimum width of the initial contact zone is 0 (line to surface contact), the corresponding fretting stress *σ_fretting_* value is 398.3 MPa, which is 23 MPa larger than the corresponding fretting stress value when the width of the contact area is 4.095 mm.

It appears from the results of the analysis that the change of contact zone width of dovetail model will not affect the stress distribution and the position of dangerous points of the structure under the fretting load. However, with the decrease in contact zone width, the contact stress value will decrease, the fretting stress value will also decrease accordingly, and the fretting life of the sample will be longer. It can also be seen from the basic Hertz theory that the contact stress value will decrease as the fillet angle increases and the contact zone decreases. To sum up, on the premise of meeting the assembly requirements and saving costs, the processing of structural parts in actual projects should focus on the width of the contact zone, which should be less than 4.3 mm. Therefore, only a pair of reduced dovetail models are taken as the research object, and more conditions, such as applicable location, environment, structural strength requirements, etc., need to be comprehensively considered to determine the accurate range of the selected contact zone width, so as to narrow the optimal range of the contact zone width.

### 4.4. Friction Coefficient of Contact Surface

At present, for the fretting fatigue problem of standard parts, all theoretical models for solving contact stress are established without considering the coupling effect of axial load and normal load. However, with the application of cyclic load, the maximum contact stress between the fretting pad and the standard sample changes with the effect of friction, not a fixed value. Under a certain load, the difference of friction coefficient between the fretting pad and the standard sample will affect the magnitude of friction work and the magnitude of contact stress. This paper uses ABAQUS to study and analyses the effect of friction factors on fretting fatigue.

The peak cyclic axial load of 1.13 × 10^4^ N was kept constant and the friction coefficients were set to 0.3, 0.4, 0.5, 0.6, 0.7, and 0.8. The dovetail model loading was numerically analyzed in order to analyze the effects of contact stress, Mises equivalent force S, and the friction stress of the *σ_fretting_* friction coefficient on the friction fatigue between the dovetail models with different friction coefficients.

**Figure 18:**
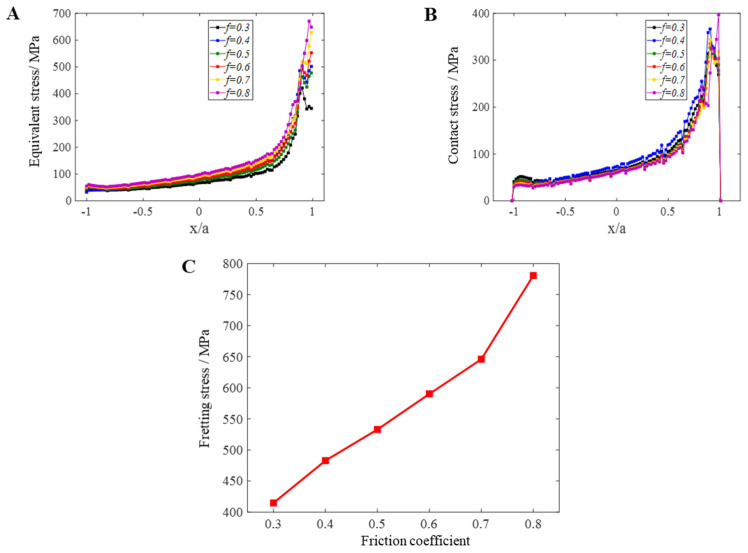
Comparison of fretting stress of the different friction coefficient models under the same load conditions: (**A**) Mises equivalent stress S value with different friction coefficients; (**B**) contact stress value with different friction coefficients; (**C**) comparison of fretting stresses of different friction coefficient models under the same load conditions.

Figure 18 only lists the Mises equivalent stress nephogram and contact stress nephogram of dovetail model when the friction coefficient is 0.8 and 0.7, but not all models. This is because qualitatively speaking, as long as it involves plane contact, there is not much difference in its stress distribution subjected to fretting load. The contact stress and maximum Mises equivalent stress S of the mortise appear at the edge of the contact area and do not change, so only two models are shown here. Quantitatively, as the friction coefficient increases, the maximum Mises equivalent stress S, maximum contact stress, and fretting stress *σ_fretting_* values increase correspondingly, because under the same fretting load, the friction coefficient increases. With the increase in load cycles, the work carried out by the friction force increases correspondingly and the stress value also increases. This requires us to ensure the processing accuracy and assembly accuracy of the sample insofar as possible to ensure the low friction coefficient of the sample contact area, which is also true in engineering practice. In general, when keeping other parameters unchanged, the friction coefficient increases from 0.3 to 0.8, subject to fretting load; the maximum Mises equivalent stress S increases by 249.425 MPa; the maximum contact stress increases by 83.054 MPa; and the fretting stress *σ_fretting_* value increased by 366.258 MPa. Separately, with the increase in friction coefficient, the values of each parameter also increase. See Table 10 for details. It can be seen that the maximum contact stress and fretting stress increase from 0.3 to 0.7, there is little difference when the friction coefficient increases the *σ_fretting_* value of friction, but when the friction coefficient increases from 0.7 to 0.8, the maximum contact stress and fretting stress *σ_fretting_* value are increased abruptly.

From the analysis, it can be seen that the variation of the friction coefficient of the dovetail model does not affect the stress distribution of the structure and the location of the danger points under the fretting load. However, with the decrease in friction coefficient, the fretting stress value will decrease accordingly, and the fretting life of the sample will be longer. Therefore, in actual projects, the friction coefficient of the contact surface should be reduced as much as possible under the premise of saving costs and meeting work requirements and the friction coefficient shall be less than 0.8 insofar as possible.

Based on the above fretting simulation analysis of dovetail model, it can be seen that in the fretting load action, changing the parameters of contact zone will not change the overall distribution trend of the Mises equivalent stress S and contact stress, nor will it change the location of structural dangerous points (still appearing at the edge of contact area), meeting the basic characteristics of fretting fatigue, However, changing the parameters of the contact zone will seriously affect the size of the contact stress and fretting stress, thereby changing the fatigue structure strength and life. In order to prolong the fretting fatigue life of the dovetail structure, it is necessary to conduct a further comprehensive analysis to determine the optimal solution when selecting contact zone parameters.

## 5. Conclusions

In this study, a friction fatigue life prediction theoretical based on elasticity theory was established to study the influence of the plastic effect on fretting fatigue, and the maximum plastic strain range was introduced (Δ*ε*)_*p*_. A prediction model for fretting the fatigue life of the surface-to-surface contact considering the plasticity effect was proposed. The dangerous parts of the structure were evaluated according to a finite element simulation, and the fretting fatigue test was evaluated through the standard sample and dovetail sample to verify the accuracy of the model. At the same time, an ABAQUS simulation was used to carry out fretting fatigue numerical analysis on the dovetail structures with different contact forms, different contact zone widths, and different contact surface friction coefficients. With the difference in the size and distribution of contact stress, Mises equivalent stress, and displacements as a reference, the influence degree and trend of contact forms, contact zone widths, and contact surface friction coefficients on fretting fatigue were analyzed and studied and the sensitivity of factors affecting fretting fatigue was analyzed. We found that:

(1) By monitoring the strain at the lower edge of the contact area in real time, the initiation and propagation of cracks can be judged. The introduction of the maximum plastic strain improves the accuracy of the prediction of fretting fatigue life of surface-to-surface contact structures. The error between the theoretical prediction value and the test value is within 12%. The model is applicable to predicting the fretting fatigue life of structures and provides theoretical support for the design of major equipment structures.
(2) With the goal of minimizing fretting stress, a more reasonable contact form, contact zone width, and contact surface friction coefficient should be uncovered in order to reduce fretting fatigue and extend the fretting fatigue life of the model. Under the same fretting load, changing the contact zone parameters will not change the Mises equivalent force S and the contact stress profile, nor will it change the location of structural dangerous points (which still appear at the contact zone edge); the basic characteristics of fretting fatigue are thus met.
(3) Changing the parameters of the contact zone will change the magnitude of the stress, and fretting fatigue is most sensitive to the width of the contact zone and the contact form. In the actual engineering design, the size and form should be determined by combining multiple factors. For the selection of the friction coefficient, the friction coefficient shall be as small as possible in order to save costs and meet requirements.

## Figures and Tables

**Table 1:** The instanton equations parameters of nickel-based alloys.

Material Properties	A	B	C	n	m
DZ125	637	573.2	0.033	0.45	0.92

**Table 2:** Comparison of simulation and theoretical results of contact stress.

Normal Load/MPa	a/mm	Simulation/MPa	Theoretical/MPa	Error
45	2.538	824.836	750.504	9.01%
50	2.543	916.573	830.618	9.38%
60	2.548	1100.1	992.833	9.75%
70	2.554	1283.69	1161.95	9.48%
80	2.558	1467.36	1313.45	10.49%
90	2.563	1651.09	1471.87	10.85%
100	2.567	1834.9	1630.317	11.15%
110	2.572	2018.77	1786.38	11.51%
120	2.576	2202.72	1942.73	11.80%
130	2.58	2386.73	2098.11	12.09%
140	2.584	2570.81	2252.51	12.38%

**Table 3:** Material parameters of DZ125.

Material	Density *ρ* (g/cm^3^)	Elasticity Modulus E_1_ (GPa)	Poisson’s Ratio ν_1_
DZ125	8.59	183	0.41

**Table 4:** Variation of elasticity modulus during test.

Cycles	E(1 − D)/GPa	D
1	184	0
2	155	0.156
3	140	0.238
4	128	0.303
5	120	0.347
6	115	0.374
7	113	0.382
8	113	0.382

**Table 5:** Summary of fretting fatigue test data of standard specimens.

Test	Load/N	Stress Ratio ν	Frequencies/Hz	Fretting Stress/MPa	Life N/Cycles
Test-1	1.2 × 10^4^	0.1	10	534.72	936,237
Test-2	1.8 × 10^4^	0.1	10	709.94	455,875
Test-3	2.4 × 10^4^	0.1	10	873.36	52,990
Test-4	2.7 × 10^4^	0.1	10	952.07	25,160

**Table 6:** Comparison of fretting fatigue test results of standard specimens and theoretical prediction results.

Test	Axial Load *F_x_*/N	Fretting Stress *σ_fretting_*/MPa	Experimental Life *N*/Cycles	Predicted Life *N*/Cycles	Error
S2_1	1.5 × 10^4^	624.22	627,663	534,601	14.8%
S2_2	1.5 × 10^4^	655.37	590,021	515,102	12.7%
S4_1	2.1 × 10^4^	792.79	253,563	215,532	15.0%
S4_2	2.1 × 10^4^	780.34	149,639	127,983	14.5%

**Table 7:** Comparison of fretting fatigue test results and theoretical prediction results of dovetail specimens.

Test	Axial Load *F_x_*/N	Fretting Stress *σ_fretting_*/MPa	Stress Ratio R	Frequency *f*/Hz	Experimental Life *N*/Cycles	Predicted Life *N*/Cycles	Error
T1-1	8.58 × 10^3^	508.5	0.1	10	96,645	102,815	6.38%
T1-2					100,027	108,672	8.64%
T 2-1	1.18 × 10^4^	658.5			34,197	37,998	11.12%
T 2-2					33,521	36,960	10.26%
T 3-1	1.35 × 10^4^	684.1			21,545	23,653	9.78%
T 3-2					20,969	22,846	8.95%

**Table 8:** Dovetail models with different width of contact area.

Number	Tenon		Mortise		Contact Area Width/mm
	*R*_1_/mm	*R*_2_/mm	*R*_5_/mm	*R*_6_/mm	
1#	1.8	1	2	1	4.343
2#	1.8	1.1	2	1.1	4.302
3#	1.8	1.2	2	1.2	4.261
4#	1.8	1.3	2	1.3	4.219
5#	1.8	1.4	2	1.4	4.178
6#	1.8	1.5	2	1.5	4.136
7#	1.8	1.6	2	1.6	4.095

**Table 9:** Variation of parameters caused by the change of width of contact area.

Model	Reduction of Maximum Mises Equivalent Stress S/MPa	Reduction of Maximum Contact Stress/MPa	Reduction of Fretting Stress/MPa
2#–3#	26.08	186.386	333.417
3#–4#	8.827	18.176	32.514
4#–5#	20.771	20.358	36.42
5#–6#	10.464	48.092	86.03
6#–7#	9.144	35.982	64.367

**Table 10:** Variation of parameters caused by change of friction coefficients.

Friction Coefficient	Reduction of Maximum Mises Equivalent Stress S/MPa	Reduction of Maximum Contact Stress/MPa	Reduction of Fretting Stress/MPa
0.3–0.4	37.665	6.798	61.8
0.4–0.5	6.048	6.048	56.440
0.5–0.6	75.449	8.521	57.310
0.6–0.7	75.449	8.521	55.879
0.7–0.8	42.041	53.166	134.830

## Data Availability

Not applicable.
